# An Ion-Releasing Bioactive Glass Accelerates Skeletal Muscle Regeneration and Functional Recovery Following Acute Injury

**DOI:** 10.3390/jfb17100500

**Published:** 2026-10-03

**Authors:** Jacob A. Kendra, Jiwei Hao, Dillon R. Harris, Rebekah L. Blatt, Richard K. Brow, Aaron B. Morton

**Affiliations:** 1 Department of Kinesiology and Sport Management, Texas A&M University, College Station, TX 77845, USA; jkendra@tamu.edu (J.A.K.); jiweihao@tamu.edu (J.H.); dillon_harris@tamu.edu (D.R.H.); 2 Department of Materials Science & Engineering, Missouri University of Science & Technology, Rolla, MO 65409, USA; rlb3nf@mst.edu (R.L.B.); brow@mst.edu (R.K.B.)

**Keywords:** angiogenesis, bioactive glass, muscle injury, regenerative medicine, skeletal muscle

## Abstract

Bioactive glasses represent an emerging strategy for localized skeletal muscle repair. Here, we investigated whether an injectable borophosphate bioactive glass [cobalt oxide–time release ion matrix (CoO-TRIM)] can accelerate skeletal muscle regeneration and functional recovery following acute injury. Using a barium chloride (BaCl_2_) injury model, muscle function, myogenesis, angiogenesis, and inflammation were evaluated in mouse tibialis anterior (TA) and extensor digitorum longus (EDL) muscles at 8 and 14 days post-injury (dpi). Mice were randomly assigned to Untreated, Saline-treated, and CoO-TRIM-treated groups. CoO-TRIM accelerated the regenerative process, with increased myofiber size and restoration of TA contractile function to values not significantly different from Healthy muscle by 14 dpi compared to Injured Untreated and Saline-treated muscles. Vascular endothelial growth factor (VEGF) concentrations were elevated through 14 dpi following CoO-TRIM treatment and accompanied by enhanced muscle vascularity in both TA and EDL muscles. CoO-TRIM modulated the inflammatory response, with increased inflammatory signaling during active regeneration at 8 dpi, followed by an accelerated transition toward a pro-regenerative environment characterized by increased CD163^+^ M2 macrophages and sustained IL-10 signaling. In contrast, CoO-TRIM produced no detectable effects in healthy contralateral muscles. These findings support ion-releasing bioactive glass as a cell- and growth factor-free strategy for skeletal muscle repair.

## 1. Introduction

Musculoskeletal injuries represent a leading cause of morbidity and a widespread health burden. Approximately 25% of adults experience musculoskeletal injuries annually, with more than 80% of reported injuries associated with physical activity [1]. These numbers are increased in highly active populations, where musculoskeletal injuries account for nearly 50% of sports-associated injuries and affect 1.6 million U.S. warfighters annually [2,3,4]. Although skeletal muscle has a remarkable capacity to regenerate, restoration of tissue structure does not imply complete recovery of contractile function [5,6]. Skeletal muscle regeneration is tightly regulated by innate biophysical and biochemical cues operating through an integrated sequence of events [7,8]. Infiltrating neutrophils and macrophages stimulate inflammatory signaling for debris clearance and repair [9,10], angiogenesis restores the microvasculature required to meet the metabolic demands of regenerating myofibers [11,12,13], and satellite cells undergo terminal differentiation forming mature myofibers [14,15]. Disruption of these regenerative events can prolong muscle weakness and fragility while impairing long-term myofiber maturation [16,17,18,19,20]. Although cellular and molecular mechanisms regulating skeletal muscle regeneration have been widely investigated, current treatment options remain largely conservative, with few therapeutic approaches capable of accelerating skeletal muscle recovery.

Tissue engineering and biomaterial-based strategies have emerged as promising approaches to improve skeletal muscle regeneration. Organic scaffolds (i.e., hydrogels, collagen, fibrin, decellularized extracellular matrix) provide bioactive cues resembling native muscle extracellular matrix, mimicking the natural regeneration cascade [21]. Synthetic and hybrid polymers possess tunable mechanical properties and controlled degradation rates, creating effective delivery vehicles [22]. Despite these advances, previous material generations require transplanted cells or recombinant growth factors to achieve functional vascularization and myofiber maturation [23,24,25]. While transplantation of satellite cells has improved muscle repair through engraftment, cell-based therapies are limited by cell isolation, expansion, and loss of stemness [26,27,28,29]. Alternatively, bolus deliveries of growth factors, vascular endothelial growth factor (VEGF), and insulin growth factor 1 (IGF-1) enhanced skeletal muscle recovery [30]. However, limitations surrounding the short half-life and bioactivity remain. Although incorporation of VEGF and IGF-1 into biomaterial delivery vehicles improved muscle regeneration, vascularization, and muscle function [30,31,32,33], the inclusion of growth factors increases manufacturing complexity and financial cost [34]. More broadly, intrinsically therapeutic biomaterials capable of eliciting biological responses through their inherent physicochemical properties have emerged as alternatives to conventional drug- or growth factor-based delivery strategies [35].

Bioactive glasses have expanded beyond conventional silicate-based glass chemistries toward other glass-forming compositions engineered for controlled degradation, near-neutral pH, resistance to hydroxyapatite formation, and release of biologically active ions suited for soft tissue repair [36,37,38,39,40]. In skeletal muscle, Jia et al. demonstrated that an aluminoborate glass promoted angiogenesis and satellite cell activation with downstream enhanced regeneration following volumetric muscle loss, providing early evidence that non-silicate glass compositions can support repair of complex skeletal muscle injuries without the use of exogenous cells or growth factors [41]. Building on this concept, our group developed cobalt oxide–time release ion matrix (CoO-TRIM), a CoO-containing Na-Ca borophosphate glass composition whose biological activity reflects the collective ionic dissolution products. Our previous work characterized the material composition, degradation rate, and ion-release profile of CoO-TRIM, demonstrating enhanced muscle function, myofiber regeneration, and revascularization in myopathic skeletal muscle [42]. However, whether CoO-TRIM can accelerate recovery following acute skeletal muscle injury and how the material influences the local regenerative microenvironment remain unknown.

Here, we investigated the regenerative effects of CoO-TRIM following BaCl_2_-induced skeletal muscle injury. CoO-TRIM was locally administered at 3 dpi, following the principal period of acute BaCl_2_-mediated myofiber degeneration and neutrophil infiltration [43,44], to minimize potential effects on the magnitude of the initial injury. Outcomes were evaluated at 8 and 14 dpi to capture active regeneration and subsequent tissue maturation. Functional, myogenic, vascular, and inflammatory responses were assessed in the tibialis anterior (TA) and extensor digitorum longus (EDL) muscles. Circulating inflammatory markers were further evaluated to determine whether local CoO-TRIM treatment elicited a systemic inflammatory response. Our findings support CoO-TRIM as a promising approach for efficient recovery of skeletal muscle structure and function following injury.

## 2. Materials and Methods

### 2.1. Glass Fabrication

CoO-TRIM is a CoO-incorporated Na-Ca-borophosphate bioactive glass with an analyzed composition (%wt): 34.4% B_2_O_3_, 34.3% P_2_O_5_, 15.3% CaO, 11.2% Na_2_O, and 3.7% CoO. Briefly, CoO-TRIM was prepared from mixtures of reagent-grade NaPO_3_ (Shanghai Muhong Industrial Co., Ltd., Optical Grade, Shanghai, China), Ca(PO_3_)_2_ (Shanghai Muhong Industrial Co., Ltd., Optical Grade, Shanghai, China), H_3_BO_3_ (Fisher, ≥99.5%, Waltham, MA, USA), Na_2_CO_3_ (Alfa Aesar, 99.5%, Ward Hill, MA, USA), CaCO_3_ (Fisher, >98.0%, Waltham, MA, USA), and CoO (Sigma Aldrich, 99.5%, St. Louis, MO, USA) as raw materials using conventional glass processing methods. Fabrication methodology and dissolution kinetics (ion-release profile, phosphate anion distributions, pH) for CoO-TRIM are previously described in detail [42].

### 2.2. Ethical Approval

All protocols in this study were approved by the Animal Care and Use Committees at Texas A&M University (AUP 2022-0215). Animal care was in accordance with the National Research Council’s *Guide for the Care and Use of Laboratory Animals* (Eighth Edition, 2011).

### 2.3. Animals

**Figure 1:**
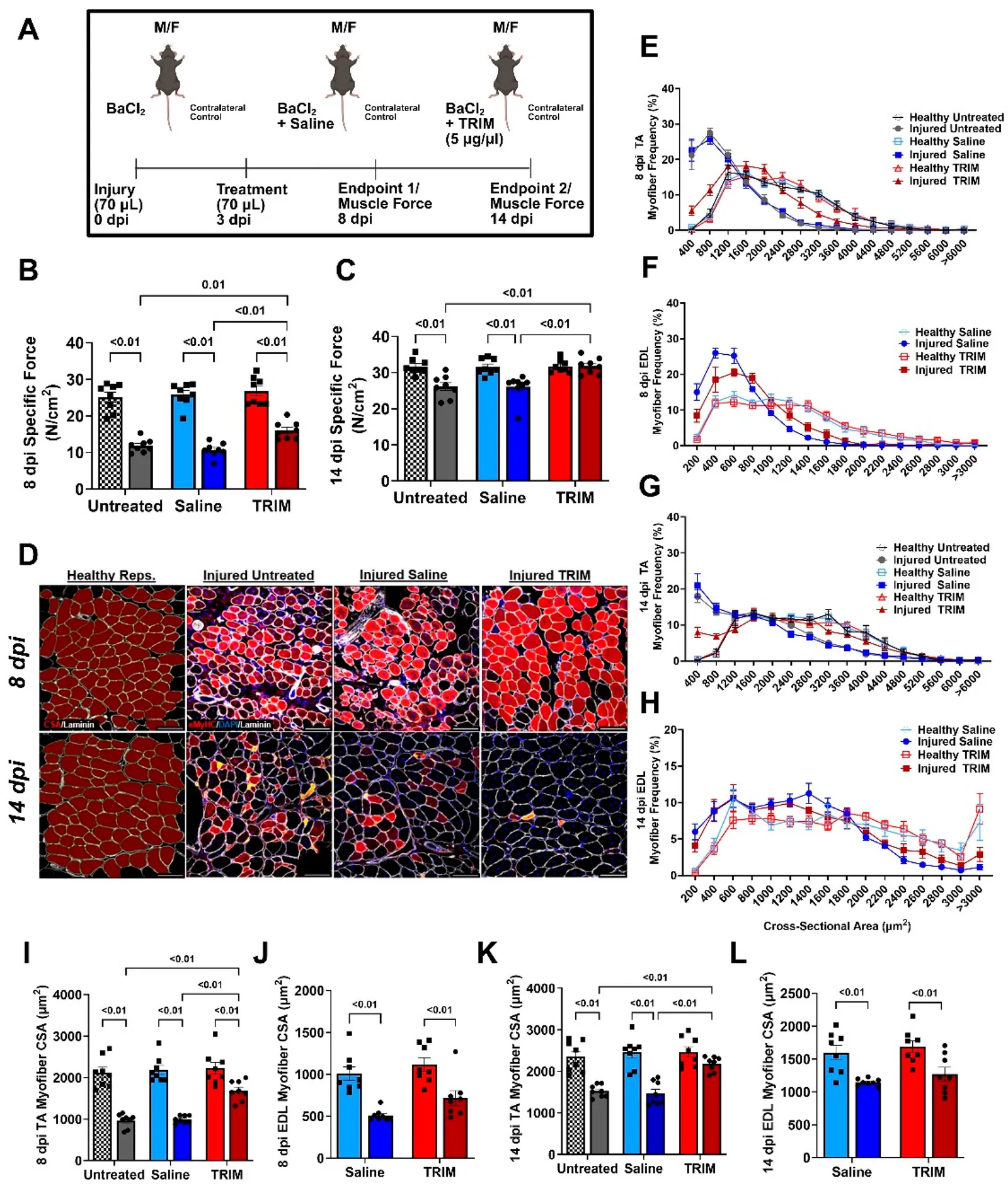
CoO-TRIM treatment accelerated functional restoration and myofiber size following injury. (**A**) Graphical experimental design. Summary values for maximal specific force via direct muscle stimulation at 120 Hz at (**B**) 8 dpi (*n* = 8) and (**C**) 14 dpi (*n* = 8). (**D**) Representative images of TA muscle cross-sections. Left column: representative images for all healthy control TA muscles. Top row: 8 dpi and bottom row: 14 dpi. Laminin (white) identified basal laminae surrounding myofibers, which were analyzed for CSA (red). Injured representative images stained for Laminin. (white) eMyHC (red) identified actively regenerating myofibers, and DAPI (blue) identified nuclei. For Injured TA muscles, only eMyHC*^+^* and CLN*^+^* fibers were analyzed compared to healthy groups. (**E**–**H**) Myofiber CSA frequency distribution for TA muscles at 8 dpi (*n* = 8) and 14 dpi (*n* = 8); EDL muscles at 8 dpi (*n* = 8) and 14 dpi (*n* = 8). (**I**–**L**) Summary values for mean TA myofiber CSA at 8 dpi (*n* = 8) and 14 dpi (*n* = 8); EDL myofiber CSA at 8 dpi (*n* = 8) and 14 dpi (*n* = 8). Summary values are means ± SEM. Comparisons were made by two-way repeated measures ANOVA; *p* < 0.05 = significant. Scale bars = 100 µm.

Male and female C57BL/6J mice (*n* = 4 males and 4 females/group) (RRID: IMSR_JAX:013141, Strain 013141, Jackson Labs; Bar Harbor, ME, USA) were housed in the Texas A&M University animal care facilities and studied at 4–5 months of age. Mice were housed on a 12 h:12 h light: dark cycle at ~23 °C, with fresh water and food available ad libitum. Mice were randomly assigned to one of three experimental groups: (1) Untreated; (2) Saline-treated (vehicle control); and (3) CoO-TRIM-treated. To induce muscle injury in vivo, mice were anesthetized via isoflurane inhalation (4% induction, 2% maintenance), the skin was shaved over the anterior hindlimbs, and 1.2% BaCl_2_ (70 µL) was injected unilaterally into either the right or left tibialis anterior (TA) muscle, with the uninjured limb serving as a contralateral control. Mice were kept warm during recovery and then returned to their cage, followed by 3 days of observation. Mice in the Saline and CoO-TRIM experimental groups were anesthetized (as above), and 70 µL of saline or 350 µg of CoO-TRIM suspended in 70 µL of saline (5 µg/µL) was injected into the TA muscle of both hindlimbs. The CoO-TRIM concentration (5 µg/µL) was maintained from our previous myopathy study [42], while the injection volume was increased to 70 µL to match the volume used for BaCl_2_ injury. At 8 and 14 dpi, both TA muscles were prepared for in situ measurements of maximal isometric force as the criterion for muscle function (Figure 1A). An additional cohort of mice (*n* = 4/group) underwent the same in situ TA muscle function assessment at 21 dpi to evaluate duration of functional benefits. No histological or biochemical analyses were performed at this time point. The mouse was then euthanized via anesthetic overdose and cervical dislocation. TA and extensor digitorum longus (EDL) muscles from both hindlimbs were removed and stored at −80 °C for histological and biochemical analyses. Serum was collected and stored at −80 °C for systemic inflammatory profiling. Investigators performing force testing and image analysis were blinded to animal treatment assignment.

### 2.4. Contractile Force

Contractile properties of the mouse TA muscles were evaluated in situ in anesthetized mice (1301A 3-in-1 Whole Animal System for Mice, Aurora Scientific, ON, Canada). Mice were anesthetized (as above) and weighed. The distal tendon of the TA muscle was isolated, secured with a 2–0 suture, and then severed from its insertion. The mouse was placed prone on a platform set to 37 °C using a heated water pump; a heat lamp maintained the muscle at 32 °C. A vertical metal peg was inserted into the patellar tendon to immobilize the knee, while the TA tendon was attached to a lever arm with a micrometer to adjust the optimal length (L_o_), as determined during twitch contractions at 1 Hz. A strip of KimWipe^®^ was wrapped around the exposed TA muscles and irrigated with sterile saline warmed to 37 °C. For direct field stimulation, a pair of platinum/iridium wire electrodes was placed across the TA muscle belly to generate sufficient current to depolarize muscle fibers. Maximum tetanic contractions were obtained at 120 Hz for 500 ms. Data for force production was acquired using manufacturer software (610A Dynamic Muscle Control/Analysis, Aurora Scientific, ON, Canada) on a personal computer. Following contractions, the TA and EDL muscles were removed, blotted of excess moisture, and weighed (ME54E, Mettler Toledo; Columbus, OH, USA). To define specific force (N/cm^2^), muscle cross-sectional area (CSA) was calculated as: CSA = muscle mass (mg)/[muscle length at L_o_ (mm) × muscle density (1.06 mg mm^−3^)] [45,46]. Maximal specific force (N/cm^2^) was calculated by dividing maximal tetanic force by muscle CSA following appropriate unit conversion.

### 2.5. Immunofluorescence

Primary antibodies used were mouse anti-embryonic myosin heavy chain (1:5, RRID: AB_528358, Cat. # F1.652, Developmental Studies Hybridoma Bank, The University of Iowa Department of Biology; Iowa City, IA, USA), rat anti-CD31 (1:200, RRID: AB_393571, BD Biosciences, Franklin Lakes, NJ, USA), mouse anti-CD163 (ED2) (1:100, RRID: AB_2260022, Cat. # sc-58965, Santa Cruz Biotechnology, Santa Cruz, CA, USA), mouse anti-iNOS (1:100, RRID: AB_627810, Cat. # sc-7271, Santa Cruz Biotechnology, Santa Cruz, CA, USA), rat anti-F4/80 (1:100, RRID: AB_323279, Cat. #MCA497R, Bio-Rad, Hercules, CA, USA), and rabbit anti-laminin (1:400, Cat.# NC1732938, Fisher Scientific; Hampton, NJ, USA). Secondary antibodies were all from Fisher Scientific (Hampton, NJ, USA): Alexa Fluor 488 Goat anti-mouse (1:200 or 1:400, RRID: AB_2633275, Cat.# PIA32723), Alexa Fluor 647 Goat anti-rat (1:200 or 1:400, RRID: AB_2895299, Cat.# A48265), and Goat anti-rabbit Rhodamine (TRITC) (1:400, RRID: AB_90296, Cat.# AP132RMI).

Mouse TA and EDL muscles were embedded in Tissue-Plus^TM^ O.C.T. Compound (Scigen, Fischer Scientific, Hampton, NJ, USA), frozen in liquid nitrogen-cooled 2-methyl butane (Thermo Fisher Scientific, Waltham, MA, USA), and sectioned (thickness, 10 μm) in a Cryostar NX50 Cryostat (Epredia, Kalamazoo, MI, USA) at −17 °C onto a microscope slide. Sections were permeabilized in 0.5% Triton X-100, washed in Tris-buffered saline (TBS), fixed with either ice-cold 4% paraformaldehyde or acetone for 10 min, washed 3× in TBS, and then blocked with 3% bovine serum albumin (BSA)/5% normal goat serum (NGS)/3% Triton X-100 for 1 h at room temperature (RT). Primary antibodies were incubated for 60–90 min at RT in blocking buffer (as above). Sections were washed 3× in TBS, incubated with secondary antibodies in blocking buffer for 60 min at RT, washed 3× in TBS, and mounted in Invitrogen^TM^ ProLong^TM^ Gold antifade reagent with DAPI (Cat.# P36941, Fisher Scientific, Hampton, NJ, USA). Slides were imaged on a Stellaris 5 White Light Laser confocal microscope (Leica Microsystems, Deer Park, IL, USA) using Leica LAS_X software (v.4.5.0.25531, RRID: SCR_013673, Leica Microsystems, Deer Park, IL, USA).

For embryonic myosin heavy chain (eMyHC^+^) analysis, 10× (N.A., 0.40) tile square images were acquired and merged in Leica LAS_X software (RRID: SCR_013673, Leica Microsystems, Deer Park, IL, USA). Using NIH ImageJ (v1.54d, RRID: SCR_003070, NIH, Bethesda, MD, USA), the total number of eMyHC^+^ fibers was counted and normalized to the total area (mm^2^) of the muscle cross-section as an index of the rate of regeneration. The total number of regenerated muscle fibers identified by centrally located nuclei (CLN) was counted in cross-section and normalized to the total area (mm^2^) of the muscle cross-section as an index of total muscle fiber regeneration.

For muscle fiber CSA and CD31^+^ analysis, 4–6 randomized 580 × 580 µm regions of interest (ROI) spanning the entire muscle were imaged with a 20× (N.A., 0.75) objective, and values across all regions were averaged per muscle. An average of 400 muscle fibers per TA and EDL muscle sections were evaluated. Muscle fiber CSA was analyzed via semi-automatic muscle segmentation analysis in NIS-Elements Advanced Research software (v5.42.06, RRID: SCR_014329, Nikon, Melville, NY, USA) presented as µm^2^. To evaluate CSA in the mouse injury groups, only eMyHC^+^ or CLN^+^ fibers were analyzed to assess CSA of regenerated muscle fibers compared to the healthy control group CSA. Myofibers were classified as small fibers (TA < 800 µm^2^; EDL < 400 µm^2^) or large fibers (TA > 2000 µm^2^; EDL > 1000 µm^2^). The total area of CD31^+^ staining per fiber was measured to evaluate microvascular area using Aivia machine learning software (v.4.5.0.25531, RRID: SCR_013673, Leica LAS_X Software imported calibration: 0.57 µm/px) (v10.5, Aivia, Bellevue, WA, USA) and normalized to the total number of muscle fibers in the ROI’s stated above. For the endothelial cell (EC)-to-myofiber ratio, the number of CD31^+^ puncta was divided by the number of myofibers in each ROI.

For macrophage phenotype analysis, 3–4 randomized 580 × 580 µm ROIs were imaged with a 20× (N.A., 0.75) objective, and values across all regions were averaged per muscle. Macrophages were identified by F4/80^+^ staining and phenotypes classified as iNOS^+^ (M1 macrophage) or CD163^+^ (M2 macrophage). Using NIH ImageJ (v1.54d, RRID: SCR_003070, NIH, Bethesda, MD, USA), images were split by color channel and converted to 8-bit for thresholding to highlight the positively stained areas of each of the 3 classifications: Pan, M1, and M2 macrophages. The area of ROI occupied by iNOS^+^ or CD163^+^ staining was divided by the area of F4/80^+^ staining × 100 to assess iNOS:F4/80 and CD163:F4/80 macrophage ratios. The M2/M1 macrophage ratio was calculated as: the CD163-to-F4/80 ratio divided by the iNOS-to-F4/80 ratio (CD163:iNOS).

### 2.6. Muscle Tissue Preparation

TA muscle samples frozen for biochemical analyses were homogenized in lysis buffer (pH = 7.4) containing 5 mM Tris-HCI and 5 mM EDTA with protease and phosphatase inhibitors (Sigma-Aldrich, St. Louis, MO, USA). Homogenates were centrifuged at 1500× *g* for 10 min at 4 °C. The supernatant (soluble fraction) was aspirated, and its protein concentration assessed using the Bradford method (Sigma-Aldrich, St. Louis, MO, USA).

### 2.7. Western Blot

All primary and secondary antibodies were used at a 1:500 and 1:20,000 concentration, respectively. Membranes were scanned with a Li-Cor Odyssey DLx Imager (Li-Cor Biotechnology, Lincoln, NE, USA) and analyzed using Image Studio Lite. Primary antibodies of interest were Pax7 (RRID: AB_528428, Cat. # PAX7, Developmental Studies Hybridoma Bank, The University of Iowa Department of Biology; Iowa City, IA, USA), MyoD (G-1) (RRID: AB_2813894, Cat. # sc-377460, Santa Cruz Biotechnology, Santa Cruz, CA, USA), and Myogenin (F5D) (RRID: AB_627980, Cat. # sc-377460, Santa Cruz Biotechnology, Santa Cruz, CA, USA).

Protein concentration of each sample was normalized in 4× Laemmli sample buffer (Cat. # 1610747, Bio-Rad, Hercules, CA, USA) containing 5% dithiothreitol. Samples were loaded on 4–20% gradient Criterion TGX Midi-Protein gels (Cat. # 5678095, Bio-Rad, Hercules, CA, USA) for electrophoresis and transferred to LF-PVDF membranes (Cat. # IPFL00010, Millipore, Burlington, MA, USA). Following transfer, membranes were blocked in either 5% milk solution or Intercept TBS Blocking Buffer (Li-Cor Biotechnology, Lincoln, NE, USA) for 1 h at RT; followed by incubation with primary antibodies overnight at 4 °C. Membranes were exposed to either IRDye 680RD Goat anti-Mouse IgG (RRID: AB_10956588, Li-Cor Biotechnology, Lincoln, NE, USA) or IRDye 800CW Goat anti-Rabbit IgG secondary antibodies (RRID: AB_621843, Li-Cor Biotechnology, Lincoln, NE, USA) for 1 h at RT. Western blots were normalized to total protein according to manufacturer recommendations for fluorescent western blotting using Revert Total Protein Stain (Cat. # 92611021, Li-Cor Biotechnology, Lincoln, NE, USA). Individual data points are displayed as fold change (FC) of corresponding Healthy Saline means. Membranes and total protein stain images are included in the data supplement.

### 2.8. ELISA

Samples of mouse TA muscle supernatant (50 µL sample; referred to above) were analyzed using a quantikine enzyme-linked immunosorbent assay (ELISA) to determine VEGFA (RRID: AB_2847842, Cat. # MMV00, R&D Systems, Minneapolis, MN, USA) protein concentrations according to the manufacturer’s instructions. Additional analyses were performed to determine cytokine and chemokine concentrations in TA muscle supernatant and serum using V-Plex Pro-inflammatory Panel 1 (mouse) kits (Cat.# K15048D-1, Meso Scale Discovery, Rockville, MD, USA) according to the manufacturer’s instructions for the following analytes: IFN-y, IL-4, IL-5, IL-6, IL-10, IL-12p70, KC/GRO (CXCL1), and TNF-α. Concentrations for VEGFA and chemokine/cytokines were determined by comparing samples to respective recombinant protein standards supplied by the manufacturer. For VEGFA, analyte concentrations were normalized to individual sample protein concentration assessed using the Bradford method (Sigma-Aldrich, St. Louis, MO, USA). For pro-inflammatory panel chemokine/cytokine concentrations, muscle supernatant protein levels were assessed using the Bradford method and diluted to equal concentrations prior to loading.

### 2.9. Statistics

Summary data are reported as means ± standard error of the mean (SEM). Statistical analyses were performed using GraphPad Prism (v10.6, RRID: SCR_002798, GraphPad Software, La Jolla, CA, USA). Data were analyzed using multiple two-tailed *t*-tests with Holm–Sidak’s correction for multiple comparisons, one-way ANOVA (Treatment), or two-way repeated measures ANOVA with Condition as the within-subject factor and Treatment as the between-subject factor, as appropriate. When unequal sample sizes or missing matched values precluded repeated measures analysis, a mixed-effects model was used. Holm–Sidak multiple-comparisons were performed when appropriate. Values for “*n*” refer to the number of animals studied in each experimental group. A power analysis for mouse experiments was conducted a priori, indicating that a minimum of *n* = 6 was sufficient to reach a power of 0.8. Cytokine analysis included *n* = 6–8/group, with the exception of serum IFNγ (*n* = 5–7/group), serum IL-4 (*n* = 2–6/group), and serum IL-6 (*n* = 5/group) due to failed assay wells; these individual outcome measures were, therefore, interpreted with appropriate caution. Sex was not included as a biological variable for the present study. *p* ≤ 0.05 was considered statistically significant.

## 3. Results

### 3.1. Functional and Structural Muscle Recovery Is Accelerated Following CoO-TRIM Treatment

Maximal specific force was measured as a criterion of muscle function in mice at 8 and 14 dpi (Figure 1B,C). No changes were observed in muscle function between Healthy TA muscles at either timepoint. There were no differences at 8 or 14 dpi between Injured Untreated TA muscles and Injured Saline-treated TA muscles. While force was diminished amongst the Injured TA muscles compared to Healthy TA muscles at 8 dpi, Injured CoO-TRIM-treated TA muscle forces were greater by 35% and 49% vs. Injured Untreated (*p* = 0.013) and Injured Saline (*p* = 0.002)-treated TA muscles, respectively (Condition, *p* < 0.001; Treatment, *p* = 0.007; Interaction, *p* = 0.157) (Figure 1B). At 14 dpi, significant losses in force remained within Injured Untreated and Injured Saline-treated TA muscles compared to Healthy TA muscles, whereas specific force in Injured CoO-TRIM-treated TA muscles was restored to values not significantly different from Healthy TA muscles, including 22% greater force vs. Injured Untreated (*p* < 0.001) and Injured Saline-treated (*p* < 0.001) TA muscles, respectively (Condition, *p* < 0.001; Treatment, *p* = 0.004; Interaction, *p* = 0.011) (Figure 1C). By 21 dpi, maximal specific force in all Injured groups had recovered to values not statistically different from Healthy TA muscle, irrespective of treatment.

To assess myofiber size (Figure 1D), mouse TA myofiber CSA was partitioned into 400 µm^2^ increments and EDL myofiber CSA into 200 µm^2^ increments to create relative frequency histogram plots (Figure 1E–H). No changes were observed between Healthy TA muscles at 8 and 14 dpi in average myofiber CSA, frequency distribution, and percentage of small and large myofibers (Figure 1D–L & Supplementary Figure S1). At 8 dpi, all Injured TA and EDL muscles had a significant loss in myofiber size compared to Healthy contralateral muscles (Figure 1). While there were no differences in myofiber size 8 dpi between Injured Untreated and Injured Saline-treated TA muscles, the myofibers in CoO-TRIM-treated TA muscles were greater by 74% vs. myofibers found in Injured Untreated (*p* < 0.001) TA muscles and 70% vs. myofibers found in Injured Saline (*p* < 0.001)-treated TA muscles (Condition, *p* < 0.001; Treatment, *p* < 0.001; Interaction, *p* = 0.011) (Figure 1). The greater myofiber CSA was evident in the frequency distribution showing a rightward shift in myofiber size vs. Injured Untreated and Injured Saline-treated TA muscles (Figure 1), with a reduction in the percentage of small-area myofibers (<800 µm^2^) and an increase in the percentage of large-area myofibers (>2000 µm^2^) (Supplementary Figure S1A,B,E,F). At 8 dpi, Injured CoO-TRIM-treated EDL muscles had a significant reduction in the percentage of small-area myofibers and were 41% larger than Injured Saline-treated EDL muscles but did not reach statistical significance (*p* = 0.10, Figure 1J & Supplementary Figure S1C,D). By 14 dpi, myofiber CSA of Injured CoO-TRIM-treated TA muscles recovered to Healthy TA muscle sizes with no differences compared to Healthy groups, and a 43% increase in myofiber size vs. Injured Untreated (*p* < 0.001) TA muscles and a 33% increase vs. Injured Saline (*p* < 0.001)-treated TA muscles (Condition, *p* < 0.001; Treatment, *p* = 0.004; Interaction, *p* = 0.004) (Figure 1K). Alternatively, myofiber CSA for Injured EDL muscles remained significantly decreased 14 dpi compared to Healthy EDL muscles, with no differences between Injured Saline-treated EDL muscles and Injured CoO-TRIM-treated EDL muscles (Figure 1L).

### 3.2. Regenerating Myofiber Maturation Is Enhanced Following CoO-TRIM Treatment

**Figure 2:**
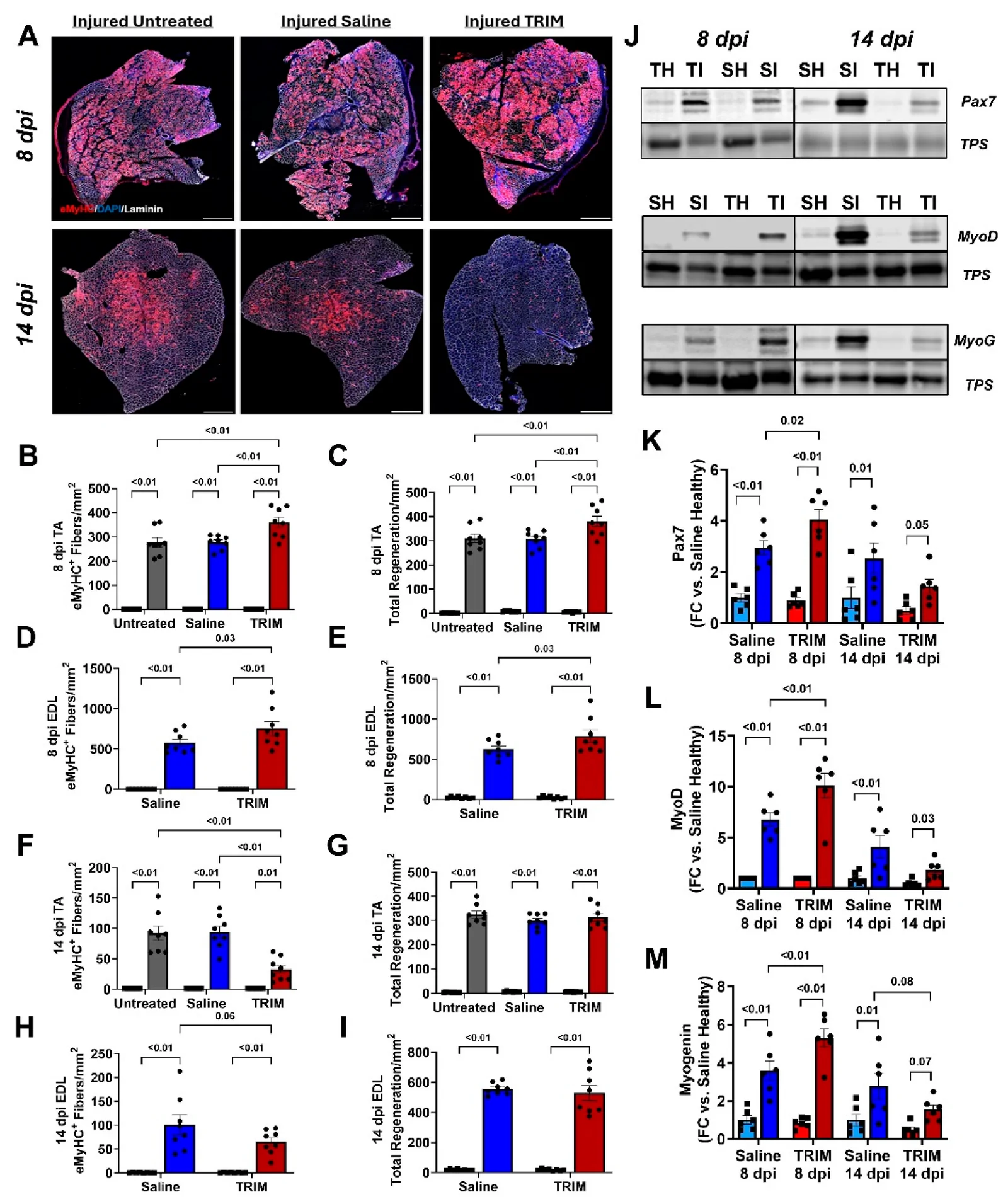
Muscle regeneration is accelerated in CoO-TRIM-treated muscles following injury. (**A**) Representative images of TA muscle cross-sections in the top row, 8 dpi, and in the bottom row, 14 dpi. Laminin (white) identified basal laminae surrounding fibers, eMyHC (red) identified actively regenerating muscle fibers, and DAPI (blue) identified nuclei. Summary values comparing (**B**,**D**) the number of eMyHC^+^ fibers (*n* = 8) and (**C**,**E**) the total number of regenerating muscle fibers (*n* = 8) (eMyHC^+^/CLN^+^ and eMyHC^−^/CLN^+^), normalized to muscle cross-section area (mm^2^) in TA and EDL muscles at 8 dpi. Summary values comparing (**F**,**H**) the number of eMyHC^+^ fibers (*n* = 8) and (**G**,**I**) the total number of regenerating muscle fibers normalized to muscle cross-section area (*n* = 8)(mm^2^) in TA and EDL muscles at 14 dpi. (**J**) Representative immunoblots and mean densitometric data for (**K**) Pax7 (*n* = 6), (**L**) MyoD (*n* = 6), and (**M**) Myogenin (*n* = 6) at 8 dpi on the left side and 14 dpi on the right side. Summary values are means ± SEM. Comparisons made by two-way repeated measures ANOVA; *p* < 0.05 = significant. Scale bars = 500 µm.

To investigate markers of regeneration, eMyHC^+^/CLN^+^ myofibers identified actively regenerating myofibers and eMyHC^−^/CLN^+^ myofibers indicated mature, regenerated myofibers (Figure 2A). As expected, Healthy TA and EDL muscles had minimal-to-no regenerated fibers following intramuscular injection into an otherwise healthy muscle (Figure 2B–I). At 8 and 14 dpi, there were no differences in actively regenerating myofibers (eMyHC^+^/CLN^+^/mm^2^) or total myofiber regeneration (eMyHC^−^/CLN^+^/mm^2^) between Injured Untreated TA muscles and Injured Saline-treated TA muscles. At 8 dpi, Injured CoO-TRIM-treated TA muscles had a 29% increase in eMyHC^+^ myofibers vs. Injured Untreated (*p* < 0.001) TA muscles and a 30% increase in eMyHC^+^ myofibers vs. Injured Saline (*p* < 0.001)-treated TA muscles (Condition, *p* < 0.001; Treatment, *p* = 0.005; Interaction, *p* = 0.005) (Figure 2B). Further analysis revealed Injured CoO-TRIM-treated TA muscles had a 25% increase in total regenerated myofibers vs. Injured Untreated (*p* < 0.001) TA muscles and a 24% increase in total regenerated myofibers vs. Injured Saline (*p* < 0.001)-treated TA muscles (Condition, *p* < 0.001; Treatment, *p* = 0.013; Interaction, *p* = 0.012) (Figure 2C). Injured CoO-TRIM-treated EDL muscles had 30% more active regenerating myofibers (*p* = 0.028; Condition, *p* < 0.001; Treatment, *p* = 0.086; Interaction, *p* = 0.086) and a 26% increase in total regenerated myofibers vs. Injured Saline (*p* = 0.03; Condition, *p* < 0.001; Treatment, *p* = 0.099; Interaction, *p* = 0.079)-treated EDL muscles (Figure 2D,E). By 14 dpi, there was a 65% reduction in actively regenerating myofibers in Injured CoO-TRIM-treated TA muscles compared to Injured Untreated (*p* < 0.001) and Injured Saline (*p* < 0.001)-treated TA muscles (Condition, *p* < 0.001; Treatment, *p* < 0.001; Interaction, *p* < 0.001) (Figure 2F) and a 36% decrease in Injured CoO-TRIM-treated EDL muscles compared to Injured Saline (*p* = 0.06)-treated EDL muscles (Figure 2H), suggesting that CoO-TRIM accelerated muscle regeneration following injury.

To further assess accelerated muscle regeneration following CoO-TRIM treatment in injured skeletal muscles, we immunoblotted prominent myogenic cell lineage markers: Pax7, MyoD, and Myogenin (MyoG) (Figure 2J). Because Injured Untreated and Injured Saline-treated TA muscles exhibited comparable functional and regenerative outcomes, subsequent Western blot analyses were focused on the Saline-treated vehicle control and CoO-TRIM-treated TA muscles. This design enabled samples for each comparison to be analyzed on the same membrane while maintaining group sample size. Concomitant with our immunofluorescence analysis, protein abundance at 8 dpi shows Injured CoO-TRIM-treated TA muscles had 37% greater abundance of Pax7 (*p* = 0.015; Condition, *p* < 0.001; Treatment, *p* = 0.134; Interaction, *p* = 0.017) (Figure 2K), 50% more MyoD (*p* = 0.005; Condition, *p* < 0.001; Treatment, *p* = 0.035; Interaction, *p* = 0.035) (Figure 2L), and 47% more MyoG (*p* = 0.008; Condition, *p* < 0.001; Treatment, *p* = 0.09; Interaction, *p* = 0.017) (Figure 2M) compared to Injured Saline-treated TA muscles. As indicated by the diminished population of eMyHC^+^ myofibers in Injured CoO-TRIM-treated TA muscles at 14 dpi, there was a 43% reduction in Pax7 abundance (*p* = 0.135) (Figure 2K), a 56% decrease in MyoD (*p* = 0.026; Condition, *p* = 0.02; Treatment, *p* = 0.057; Interaction, *p* = 0.125) (Figure 2L), and a 45% loss of MyoG (*p* = 0.08) (Figure 2M) compared to Injured Saline-treated TA muscles as myogenic signaling returns to near basal levels with myofibers completing the regenerative process.

### 3.3. CoO-TRIM Treatment Stimulates Angiogenic Growth Factors and Revascularization

**Figure 3:**
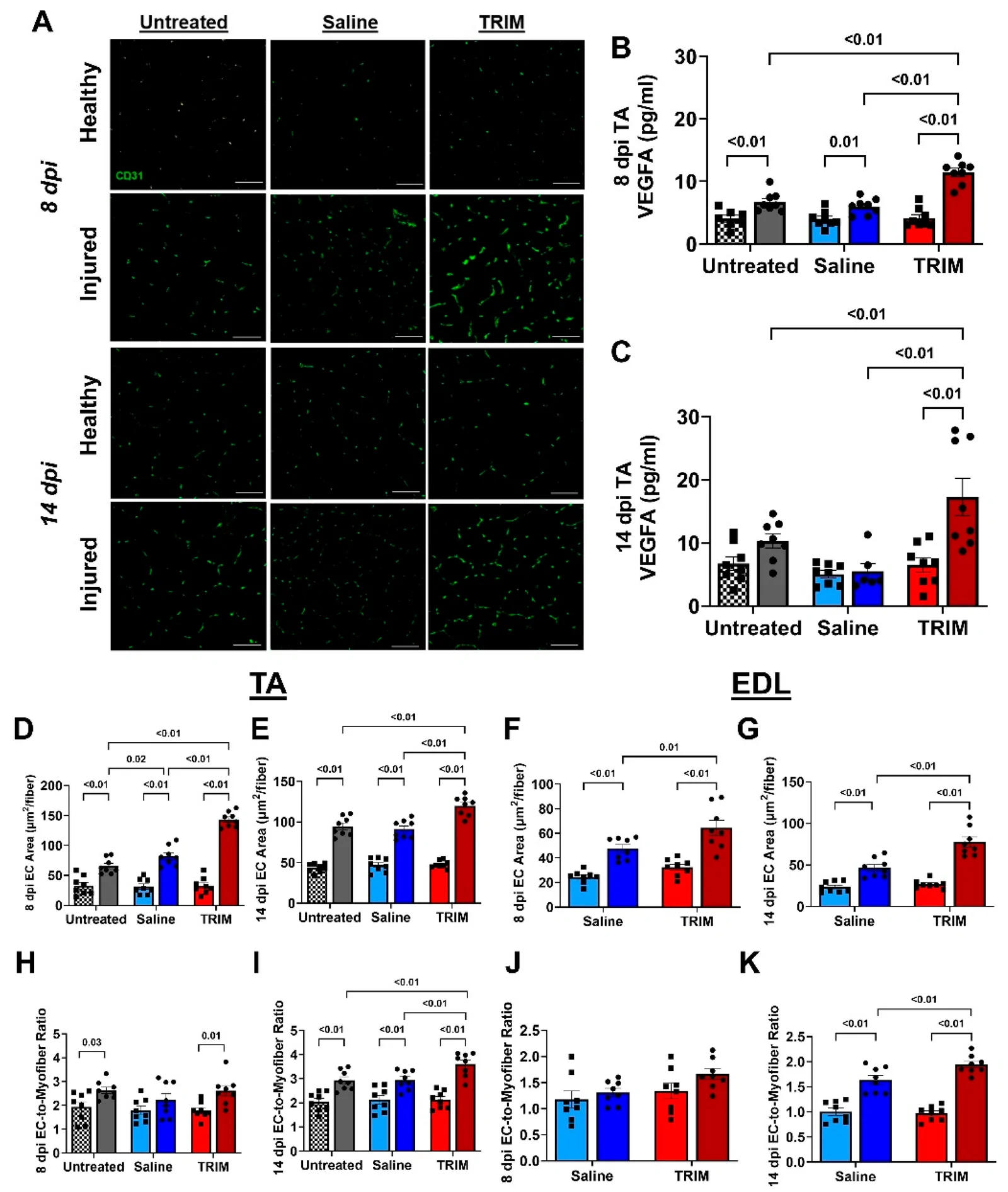
CoO-TRIM increases VEGF production and muscle vascularity. (**A**) Representative images of TA muscle cross-sections at 8 dpi on the top and 14 dpi on the bottom. CD31+ immunostaining (green) identifies endothelial cells (ECs) as a measure of vascularity. Summary values for VEGFA concentrations in whole muscle TA homogenates probed by ELISA at (**B**) 8 dpi (*n* = 7–8) and (**C**) 14 dpi (*n* = 6–8). Summary values for EC area in (**D**,**E**) TA muscle cross-sections (*n* = 8) and (**F**,**G**) EDL muscle cross-sections (*n* = 8) at 8 and 14 dpi. Summary values for the EC-to-myofiber ratio in (**H**,**I**) TA muscle cross-sections (*n* = 8) and (**J**,**K**) EDL muscle cross-sections (*n* = 8) at 8 and 14 dpi. Summary values are means ± SEM (*n* = 6–8/group). Comparisons made by two-way repeated measures ANOVA or mixed-effects model; *p* < 0.05 = significant. Scale bars = 100 µm.

VEGF is a well-defined regulator of regeneration, with synergistic effects on satellite cell proliferation and angiogenesis [47,48,49]. The ions released from CoO-TRIM have previously demonstrated increased VEGF production and angiogenesis in myopathic skeletal muscle [42], but the ability to stimulate growth factor release and revascularization in Injured skeletal muscle is unknown. At 8 and 14 dpi, no differences were observed between Healthy TA muscles irrespective of treatment (Figure 3B,C). While VEGF concentrations were increased in all Injured TA muscles compared to Healthy TA muscles, Injured CoO-TRIM-treated TAs experienced a near 2-fold increase in VEGF concentration vs. Injured Untreated (*p* < 0.001) and Injured Saline (*p* < 0.001)-treated TA muscles (Condition, *p* < 0.001; Treatment, *p* < 0.001; Interaction, *p* < 0.001) (Figure 3B). With increased time for matrix degradation and ion release, VEGF concentration was further elevated at 14 dpi in Injured CoO-TRIM-treated TA muscles compared to Healthy TA muscles (*p* < 0.001) and Injured Untreated (*p* = 0.005) and Injured Saline-treated (*p* < 0.001) TA muscles (Condition, *p* < 0.001; Treatment, *p* = 0.005; Interaction, *p* = 0.003) (Figure 3C). The absence of VEGF stimulation in Healthy CoO-TRIM-treated TAs suggests that the ions released modulate upstream regulators of VEGF that are activated within the injury microenvironment rather than direct stimulation of VEGF.

To determine whether the injury-dependent increase in VEGF was accompanied by enhanced revascularization, we evaluated CD31 as a biomarker of ECs and vascularity [50]. As expected, EC area was increased in Injured Untreated, Injured Saline-treated, and Injured CoO-TRIM-treated TA muscles compared to their respective Healthy contralateral controls at 8 dpi (Figure 3D), which was consistent with an early vascular response to injury [11,13]. By 14 dpi, both EC area and the EC-to-myofiber ratio were enhanced across all Injured groups (Figure 3E,I), indicating continued revascularization throughout the regeneration process. Importantly, Injured CoO-TRIM-treated TA muscles exhibited greater EC area at 8 (Condition, *p* < 0.001; Treatment, *p* < 0.001; Interaction, *p* < 0.001) and 14 dpi (Condition, *p* < 0.001; Treatment, *p* < 0.001; Interaction, *p* < 0.001) (Figure 3D,E) and an increased EC-to-myofiber ratio at 14 dpi compared to Injured Untreated (*p* = 0.009) and Injured Saline (*p* = 0.009)-treated TA muscles (Condition, *p* < 0.001; Treatment, *p* = 0.085; Interaction, *p* = 0.028) (Figure 3I). A similar response was observed in the EDL muscle as Injured CoO-TRIM-treated EDL muscles had enhanced vascularity at 8 (*p* = 0.008; Condition, *p* < 0.001; Treatment, *p* = 0.002; Interaction, *p* = 0.316), and 14 dpi (*p* < 0.001; Condition, *p* < 0.001; Treatment, *p* = 0.003; Interaction, *p* < 0.001) compared to Saline-treated EDL muscles (Figure 3F,G,K). Neither EC area nor the EC-to-myofiber ratio differed between Healthy contralateral TA and EDL muscles (Figure 3D–K), further supporting an injury-dependent mechanism underlying CoO-TRIM.

### 3.4. CoO-TRIM Modulates the Local Inflammatory Response During Skeletal Muscle Regeneration

**Figure 4:**
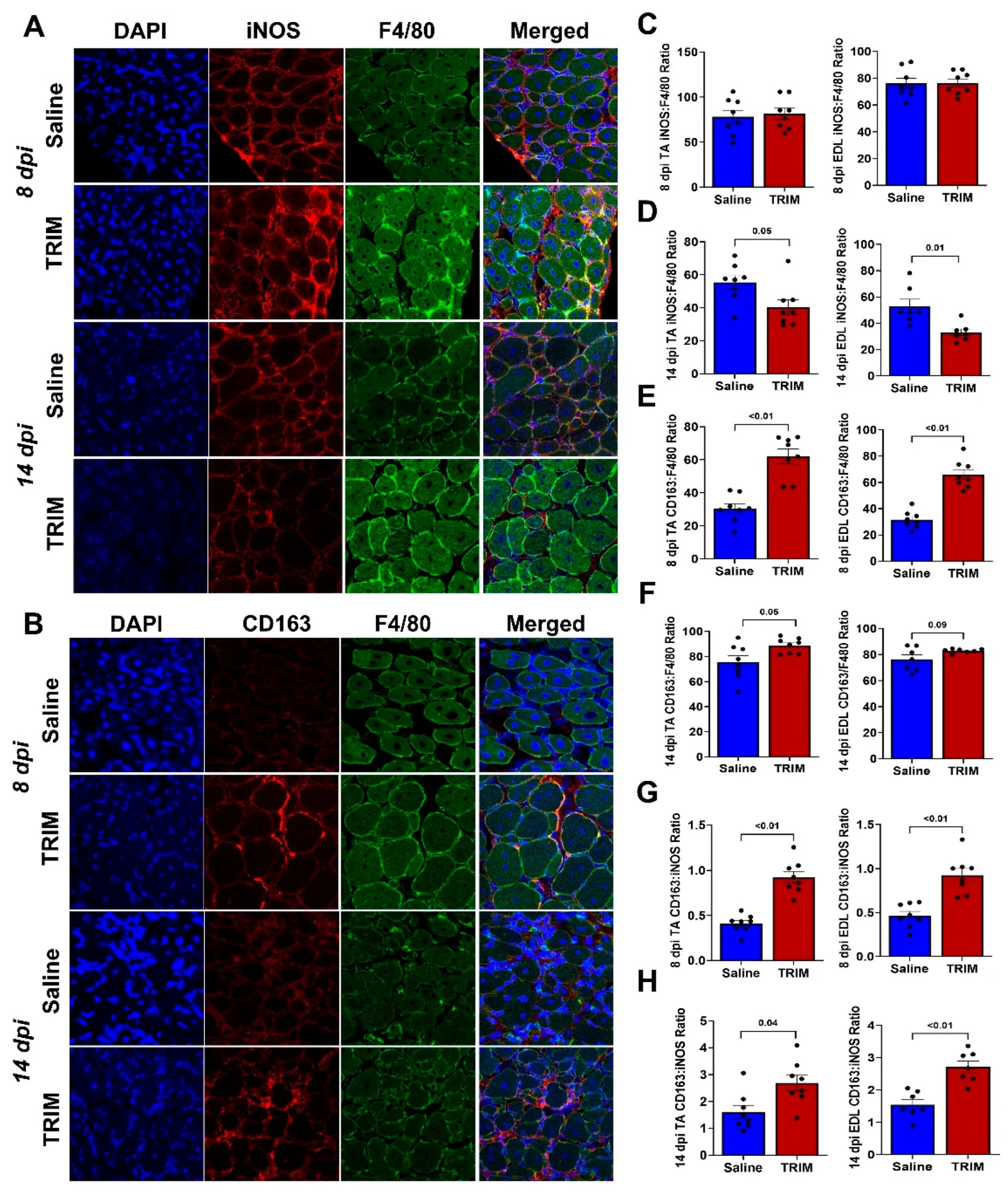
CoO-TRIM modulates macrophage polarization. Representative images of TA muscle cross-sections stained for (**A**) M1 macrophage phenotype and (**B**) M2 macrophage phenotype; DAPI (blue) identified nuclei, iNOS/CD163 (red) indicated M1 or M2 macrophage phenotype, and F4/80 (green) labeled pan-macrophages. Summary values are means ± SEM for (**C**) 8 dpi TA (*n* = 8) and EDL (*n* = 8) iNOS:F4/80 ratio; (**D**) 14 dpi TA (*n* = 8) and EDL (*n* = 7) iNOS:F4/80 ratio; (**E**) 8 dpi TA (*n* = 8) and EDL (*n* = 8) CD163:F4/80 ratio; (**F**) 14 dpi TA (*n* = 8) and EDL (*n* = 7) CD163:F4/80 ratio; (**G**) 8 dpi TA (*n* = 8) and EDL (*n* = 8) CD163:iNOS ratio; and (**H**) 14 dpi TA (*n* = 8) and EDL (*n* = 7) CD163/iNOS ratio. Comparisons made vs. Injured Saline-treated by multiple two-tailed *t*-tests with Holm–Sidak correction for multiple comparisons; adjusted *p* < 0.05 = significant. Scale bars = 100 µm.

**Figure 5:**
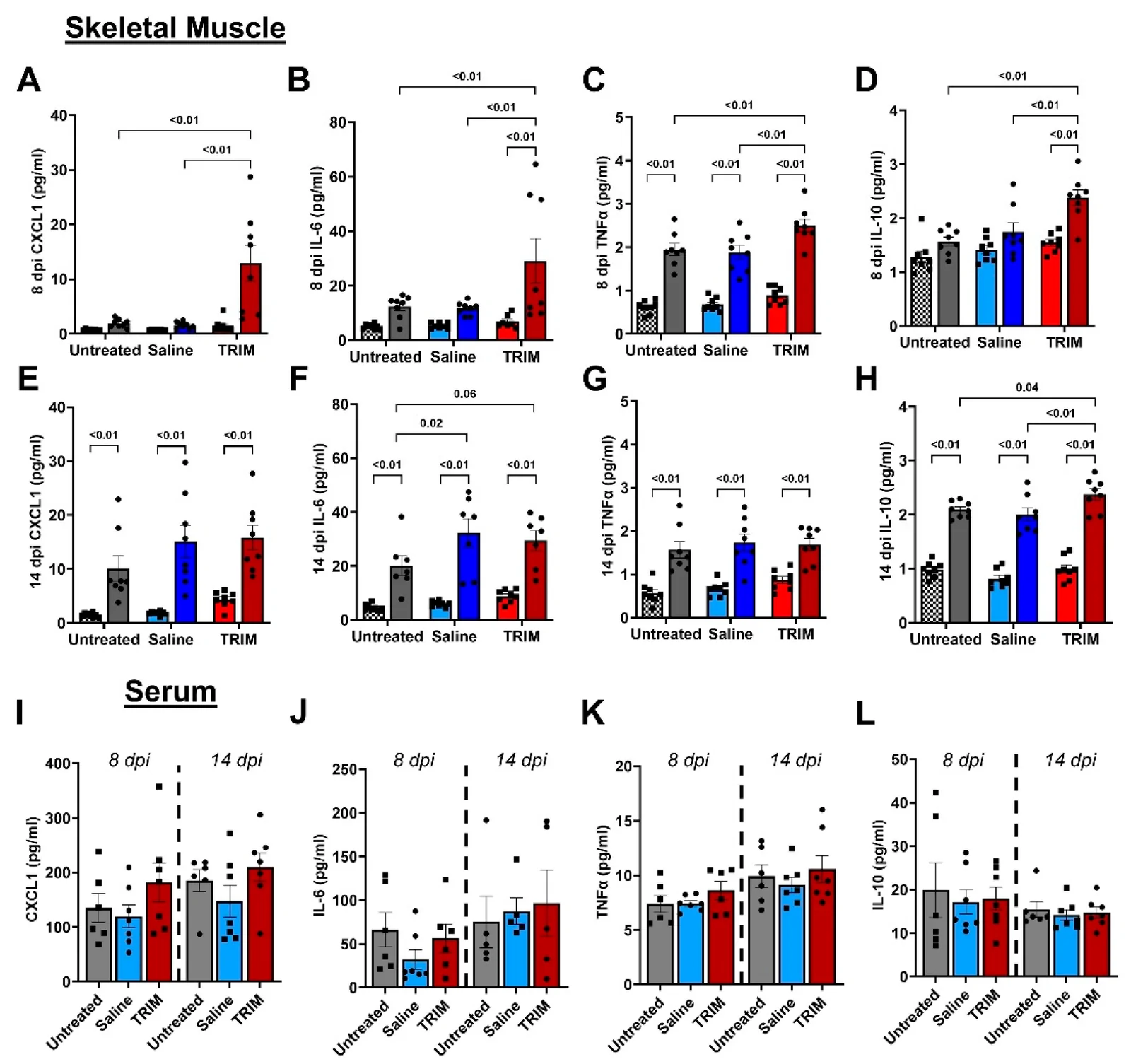
CoO-TRIM stimulates local inflammatory cytokines without systemic response. Summary values for inflammatory cytokines probed by the MSD Inflammatory Panel: CXCL1 at (**A**) 8 dpi (*n* = 8), (**E**) 14 dpi (*n* = 8), and (**I**) serum (*n* = 6–7); IL-6 at (**B**) 8 dpi (*n* = 7–8), (**F**) 14 dpi (*n* = 7–8), and (**J**) serum (*n* = 5–7); TNFα at (**C**) 8 dpi (*n* = 8), (**G**) 14 dpi (*n* = 8), and (**K**) serum (*n* = 6–7); IL-10 at (**D**) 8 dpi (*n* = 8), (**H**) 14 dpi (*n* = 8), and (**L**) serum (*n* = 6–7). Summary values are means ± SEM. Comparisons were made by one-way ANOVA (serum), two-way repeated measures ANOVA, or mixed-effects model; *p* < 0.05 = significant.

To characterize the inflammatory response associated with CoO-TRIM-mediated regeneration, we evaluated macrophage polarization (Figure 4) and pro- and anti-regenerative inflammatory cytokines (Figure 5) in skeletal muscle. Because inflammatory cytokine responses were comparable between Injured Untreated muscles and Injured Saline-treated muscles, macrophage phenotyping was subsequently focused on the Injured Saline-treated vehicle control and Injured CoO-TRIM-treated muscles. At 8 dpi, the CD163/iNOS macrophage ratio was 2-fold greater (*p* < 0.001) in Injured CoO-TRIM-treated muscles compared to Injured Saline-treated muscles (Figure 4G). This response was driven by an increased proportion of CD163^+^:F4/80^+^ M2 macrophage staining (Figure 4E), whereas the ratio of iNOS^+^:F4/80^+^ M1 macrophage staining was unchanged (Figure 4C). The comparable abundance of iNOS^+^ staining at 8 dpi suggests that CoO-TRIM did not reduce the magnitude of the BaCl_2_ injury but accelerated the transition to a pro-regenerative macrophage phenotype concomitant to ongoing inflammation. The elevated CD163:iNOS ratio persisted through 14 dpi in Injured CoO-TRIM-treated muscles compared to Injured Saline-treated muscles (*p* = 0.038) (Figure 4H). At 14 dpi, CoO-TRIM reduced the ratio of iNOS^+^:F4/80^+^ M1 macrophages (Figure 4D), while the CD163^+^:F4/80^+^ M2 macrophage ratio remained elevated in Injured CoO-TRIM-treated TA muscles (Figure 4F). A similar trend was observed in Injured CoO-TRIM-treated EDL muscles, indicating a sustained shift toward a pro-regenerative macrophage phenotype (Figure 4C–H).

Consistent with the enhanced early inflammatory response, CXCL1 (Figure 5A), IL-6 (Figure 4B), TNF-α (Figure 4C), and IL-10 (Figure 4D) concentrations were elevated in Injured CoO-TRIM-treated TA muscles compared to Healthy contralateral controls and Injured Untreated and Injured Saline-treated TA muscles at 8 dpi. TNF-α was the only inflammatory cytokine at 8 dpi that was elevated in all Injured TA muscles compared to Healthy TA muscles (Figure 4C). By 14 dpi, while CXCL1 and TNF-α were elevated in Injured TA muscles compared to Healthy TA muscles, no differences were observed between Injured groups (Figure 4E,G). At 14 dpi, IL-6 was elevated in Injured Saline-treated TA muscles (*p* = 0.018) compared to Injured Untreated TA muscles (Figure 4F). IL-10, a pro-regenerative cytokine released by intramuscular macrophages, was increased in all Injured TA muscles compared to Healthy TA muscles at 14 dpi; however, IL-10 remained elevated in Injured CoO-TRIM-treated TA muscles (vs. Injured Untreated, *p* = 0.038; vs. Injured Saline-treated, *p* = 0.007; Condition, *p* < 0.001; Treatment, *p* = 0.011; Interaction, *p* = 0.25) (Figure 4H), which was consistent with the sustained increase in the M2:M1 macrophage ratio. No differences were detected between Injured CoO-TRIM-treated TA muscles and Injured Untreated or Injured Saline-treated TA muscles in the remaining inflammatory cytokines (IFN-γ, IL-4, IL-5, IL-12p70) (Supplemental Figure S2).

Furthermore, no inflammatory response was observed following CoO-TRIM treatment in healthy mice, suggesting that CoO-TRIM may exert immunomodulatory effects dependent on the local injury environment. Despite the local inflammatory response observed in Injured CoO-TRIM-treated TA muscles, no differences were detected in circulating concentrations of the assessed cytokines between groups at 8 or 14 dpi (Figure 5I–L). These findings indicate the immunomodulatory response to CoO-TRIM remained localized to the regenerating muscle without a corresponding systemic inflammatory response.

## 4. Discussion

Skeletal muscle regeneration requires the coordinated progression of myogenesis, angiogenesis, and inflammation to restore tissue structure and contractile function following injury [51,52,53]. While previous regenerative therapies delivered exogenous cells or recombinant growth factors, bioactive materials capable of stimulating endogenous repair mechanisms represent a potential alternative due to their tunable biological activity and translational potential [41,42]. In the present study, local injection of CoO-TRIM promoted early myofiber maturation, increased VEGF production and vascular remodeling, and accelerated the transition toward a pro-regenerative inflammatory environment without suppressing the early inflammatory response. Importantly, these biological effects were restricted to Injured CoO-TRIM-treated muscles, with no comparable responses observed in healthy muscle and no corresponding increase in circulating inflammatory cytokines. Collectively, these findings suggest the regenerative effects of CoO-TRIM are associated with the collective ionic dissolution products released within the local injury microenvironment, supporting CoO-TRIM as a promising biomaterial strategy for accelerating skeletal muscle repair.

### 4.1. CoO-TRIM Accelerates Structural and Functional Recovery

Following acute injury, quiescent satellite cells progress through the myogenic cell cycle into nascent myofibers that integrate with the surrounding extracellular matrix and vascular network before normal force production can be restored [54,55]. CoO-TRIM accelerated this regenerative progression, producing larger regenerated myofibers and greater specific force during active regeneration, followed by restoration of contractile function to values not significantly different from Healthy muscle by 14 dpi. These findings extend previous reports that bioactive glasses can support skeletal muscle repair. Jia et al. showed that an aluminoborate glass promotes greater satellite cell activity and regeneration following volumetric muscle loss compared to traditional 45S5 silicate-based and 13-93B3 borate-based glasses [41]. Our previous work was the first to establish bioactive glass-mediated improvements to myofiber regeneration and muscle function in myopathic skeletal muscle [42]. Expanding on this framework, we demonstrate that a borophosphate bioactive glass, CoO-TRIM, can accelerate endogenous structural and functional recovery following acute skeletal muscle injury.

The temporal pattern of eMyHC and myogenic regulatory proteins following CoO-TRIM treatment further supports accelerated progression through the regenerative cycle rather than simply an increase in regenerative activity. Injured CoO-TRIM-treated muscles exhibited greater numbers of active regenerating and total regenerated myofibers at 8 dpi, accompanied by increased Pax7, MyoD, and Myogenin abundance. By 14 dpi, however, eMyHC^+^ myofibers and the associated myogenic proteins were reduced relative to injured controls, whereas regenerated myofiber size and contractile function were improved. Because eMyHC, Pax7, MyoD, and Myogenin are transiently associated with successive stages of satellite cell activation, differentiation, and early myofiber formation [8,14,15,56], their early elevation followed by normalization is consistent with an advanced regenerative timeline. Thus, CoO-TRIM appears to accelerate the transition from active myogenesis toward mature, force-producing myofibers rather than prolonging regenerative signaling. Regenerative effects were also observed in the underlying EDL muscle, although responses were generally more pronounced in the directly injected TA muscle. Our previous work demonstrated distribution of CoO-TRIM beyond the primary injection site into adjacent muscle [42], suggesting that local material distribution and diffusion of released ions may influence regeneration through the surrounding tissue compartment. The EDL response, therefore, supports a locally distributed biological effect rather than an effect exclusive to tissue adjacent to the injection site.

CoO-TRIM particles are altered following exposure to physiological media. The particles release soluble B_2_O_3_ and Na_2_O components and a Co-containing amorphous calcium polyphosphate phase precipitates on the particle surfaces, which slowly releases Co^2+^, Ca, and phosphate species [41]. The formation of Co-doped calcium phosphate reaction layers has been reported for other cobalt-containing bioactive glasses. Amorphous calcium phosphates are metastable phases whose composition and subsequent transformation can influence ion release kinetics and biological behavior [57,58]. Because the biological outcomes examined here occur 5–11 days after CoO-TRIM administration, they likely overlap with this secondary phase of material degradation. Whether the injury microenvironment and infiltrating immune cells modify formation or resorption of this conversion phase remains unknown; however, macrophages and multinucleated giant cells are known to participate in cell-mediated resorption of calcium phosphate biomaterials [59]. Although elevated Co^2+^ concentrations can promote oxidative stress, no overt evidence of CoO-TRIM-associated tissue injury was observed in otherwise healthy injected muscle, which exhibited minimal to no regenerative activity and no detectable functional or structural impairment. However, micro-focal necrosis at the injection site was not directly quantified in the present study and, therefore, cannot be definitively excluded.

The biological response to CoO-TRIM should be considered within the context of its multi-component composition rather than attributed to a single ion. The sodium–calcium borophosphate network releases multiple biologically active ions capable of influencing cellular signaling, angiogenesis, and myogenesis [36,60,61,62,63,64,65]. This distinguishes CoO-TRIM from regenerative strategies dependent upon delivery of a single recombinant growth factor and aligns with the broader development of ion-releasing biomaterials designed to stimulate endogenous repair pathways. Together with our previous findings in myopathic muscle, the present results suggest that controlled multi-ion delivery can promote skeletal muscle regeneration and improved muscle function across distinct pathological contexts.

### 4.2. CoO-TRIM Enhances Injury-Dependent VEGF Signaling and Revascularization

Restoration of the skeletal muscle microvasculature is integral to regeneration, providing oxygen, metabolic substrates, and paracrine signals supporting continued myofiber growth and maturation [66,67,68]. CoO-TRIM amplified the vascular response to injury, sustaining increased VEGF up to 14 dpi with a greater endothelial cell area and an endothelial cell-to-myofiber ratio. These findings are consistent with a growing body of work demonstrating that bioactive glasses, particularly mixed-network compositions, can enhance angiogenic signaling and vascular remodeling across in vitro and skeletal muscle injury and disease models [61,69,70,71,72]. The increased VEGF production and vascularity following CoO-TRIM treatment in myopathic skeletal muscle suggests that the response to CoO-TRIM is reproducible across distinct regenerative environments [42]. Furthermore, the vascular response observed in the underlying EDL muscle may similarly reflect local ion distribution of CoO-TRIM and diffusion of released ions beyond the primary TA injection site, as demonstrated in our previous myopathy study [42]. Although secondary paracrine signaling originating from the TA muscle cannot be excluded, the prior observation of CoO-TRIM distribution into adjacent muscle supports local ion diffusion as our primary hypothesis for the observed spatial effect following CoO-TRIM treatment.

Importantly, VEGF and vascular remodeling were enhanced only within injured muscle, with no comparable response following CoO-TRIM treatment in healthy muscle. The injury-dependent effect suggests that CoO-TRIM augments regenerative pathways already activated within the local microenvironment rather than constitutively inducing angiogenesis. Injury-associated hypoxia, inflammation, extracellular matrix remodeling, and increased metabolic demand each contribute to VEGF regulation and may provide a permissive environment in which released ions stimulate angiogenic signaling [47,51,73,74,75,76]. Although cobalt is commonly associated with a hypoxia mimetic [77], the present data do not establish a single upstream mechanism, and the vascular response is more appropriately considered within the context of coordinated multi-ion release from CoO-TRIM.

The vascular response further parallels the accelerated myogenic and functional restoration observed in Injured CoO-TRIM-treated muscle. Regenerating myofibers require adequate vascular support to sustain continued growth and maturation [78,79,80,81], while endothelial cells and infiltrating immune cells provide reciprocal signaling that further regulates satellite cell activity [82,83]. The concurrent enhancement of VEGF signaling, muscle vascularity, myofiber maturation, and contractile recovery supports a broader effect of CoO-TRIM on the regenerative microenvironment rather than selective stimulation of angiogenesis alone.

### 4.3. CoO-TRIM Promotes the Transition Towards a Pro-Regenerative Inflammatory Environment

Inflammation following skeletal muscle injury is a transient, dynamic response regulated by the phenotype and temporal progression of immune cells within the local microenvironment [10,52,84]. CoO-TRIM promoted an early shift toward a pro-regenerative macrophage phenotype while inflammatory signaling remained active. At 8 dpi, the iNOS^+^:F4/80^+^ macrophage staining ratio remained comparable between injured treatment groups, whereas the CD163^+^:F4/80^+^ macrophage staining ratio and CD163:iNOS ratio were increased following CoO-TRIM treatment. This response occurred alongside elevated local CXCL1, IL-6, TNF-α, and IL-10, indicating that CoO-TRIM promoted a reparative immune environment without eliminating the inflammatory signals associated with early regeneration [9,85]. By 14 dpi, the reduction in iNOS^+^ macrophage staining with sustained M2 polarization and elevated IL-10 concentrations further supports accelerated progression toward a pro-regenerative phenotype rather than broad suppression of inflammation. This distinction holds biological importance as early inflammatory macrophages contribute to necrotic tissue clearance and satellite cell activation [84,86,87]. The subsequent transition toward a pro-regenerative, M2 macrophage phenotype promotes myogenic differentiation, extracellular matrix remodeling, and angiogenic signaling [51,82,88]. The response following CoO-TRIM injection, therefore, appears to influence the timing and balance of immune cells as opposed to reduced inflammatory activity. This interpretation is further supported by the concomitant increases in VEGF, muscle vascularity, myofiber maturation, and functional restoration, all of which are influenced by macrophage-derived cytokines and growth factors. Although the present study does not establish a causal relationship between macrophage polarization and the observed regenerative outcomes, the synchronized progression from an M1 to M2 phenotype suggests that immune modulation may contribute to the regenerative microenvironment following CoO-TRIM injection.

To our knowledge, inflammatory responses following bioactive glass treatment in damaged skeletal muscles have not been directly characterized. Previous studies of bioactive glasses in skeletal muscle have largely focused on angiogenesis, myogenesis, and structural repair. While our prior CoO-TRIM study in myopathic muscle provided the only direct evidence that bioactive glass can influence inflammatory signaling in skeletal muscle, the present findings extend that observation to skeletal muscle injury, suggesting that the biological activity of ion-releasing borophosphate glasses may include immune cell regulation in addition to their established effect on angiogenic and myogenic pathways. Importantly, CoO-TRIM treatment did not induce comparable inflammatory responses in healthy muscle, further supporting an injury-dependent immunomodulatory effect. Moreover, no differences were detected in circulating inflammatory cytokines. Thus, despite the pronounced local immune response in Injured muscle, CoO-TRIM treatment was not associated with a systemic inflammatory response, suggesting that the effects remained local to the injured microenvironment.

### 4.4. Study Limitations and Future Directions

Several limitations should be considered when interpreting these findings. First, the present study was not designed to resolve the individual contribution of released ions from CoO-TRIM. The regenerative response, therefore, cannot be attributed specifically to B_2_O_3_, P_2_O_5_, CoO, CaO, or Na_2_O ions and likely reflects the collective biological activity of the degrading multi-ionic matrix. Compositionally matched controls, ion substitution, or altered ion-release kinetics are necessary to determine how individual ions contribute to the observed myogenic, vascular, and inflammatory responses. Similarly, although the effects on the regenerative triad were observed in parallel following CoO-TRIM treatment, the current data establish coordinated associations rather than direct mechanistic relationships. Pathway inhibition, immune cell manipulation, and more targeted molecular analyses are required to determine mechanisms underlying the observed accelerated recovery.

A second consideration is the potential interaction between CoO-TRIM and residual Ba^2+^ following chemical injury. CoO-TRIM was injected at 3 dpi, following the principal period of BaCl_2_-mediated myofiber degeneration, but residual tissue Ba^2+^ was not directly quantified, and a transient physicochemical interaction with CoO-TRIM cannot be excluded. Nevertheless, 8 dpi Injured CoO-TRIM-treated muscles retained an active inflammatory response and exhibited increases in myogenic activity, angiogenesis, subsequent contractile recovery, and greater evidence of myofibers undergoing active regeneration (Figure 2A,B), arguing against reduced initial injury severity as the primary explanation for the phenotype. Future studies comparing alternative injury models or direct measurement of tissue barium would provide insight into the regenerative effects of CoO-TRIM from potential interactions with the injury reagent.

Additional methodological limitations should be acknowledged. Although maximal specific force was assessed at 21 dpi and indicated that all Injured groups recovered to values not statistically different from Healthy muscle, histological and biochemical outcomes were evaluated only through 14 dpi. Therefore, the present study cannot determine whether structural, vascular, or inflammatory differences associated with CoO-TRIM persist after functional restoration is achieved. Longer-term studies incorporating these outcomes will be necessary to define the duration of the regenerative response. Macrophage classification using iNOS and CD163 provides broad inflammatory phenotypes but cannot capture the full heterogeneity and plasticity of macrophages during muscle repair. Future direct cell-level phenotyping studies are necessary to further investigate the inflammatory profile following CoO-TRIM treatment. Finally, the absence of changes in circulating inflammatory cytokines indicates that local CoO-TRIM treatment was not associated with a detectable systemic inflammatory response at the timepoints examined; however, this does not constitute a comprehensive assessment of systemic safety, biodistribution, or long-term material fate. Although our previous work demonstrated progressive loss of CoO-TRIM-associated biological effects between 70 and 140 days post-treatment [42], complete particle degradation and clearance from skeletal muscle have not been directly established. Thus, dedicated pharmacokinetic and pharmacodynamic studies are necessary to determine the in vivo timeline of material degradation, ion metabolism, and clearance, and to relate these processes to the duration of the biological response.

## 5. Conclusions

Local injection of CoO-TRIM accelerated skeletal muscle recovery following acute injury through coordinated enhancement of myogenesis, angiogenesis, and immune modulation. CoO-TRIM advanced endogenous regenerative processes, promoting early myofiber maturation and functional restoration while enhancing VEGF and transitioning toward a pro-regenerative inflammatory environment. These effects were exerted in injured tissue without detectable changes in circulating inflammatory cytokines, supporting a local biological response. Together with our previous findings in myopathic skeletal muscle, these results further support CoO-TRIM as a multi-ion-releasing bioactive glass capable of enhancing muscle repair across distinct pathological contexts. More broadly, this work highlights the potential of bioactive glass as a cell- and growth factor-free strategy to improve skeletal muscle regeneration.

## 6. Patents

The work described in this article is covered by patent application No. WO2023034523A1 [89]. A.B.M. and R.K.B. are co-inventors of CoO-TRIM.

## Data Availability

The data that support the findings of this study are available from the corresponding author upon reasonable request.

## Supplementary Materials

The following supporting information can be downloaded at https://www.mdpi.com/article/10.3390/jfb17100500/s1, Figure S1: Frequency (%) of small (<800 µm^2^) and large (>2000 µm^2^) myofibers in TA muscle at (A,B) 8 dpi (*n* = 8) and (E,F) 14 dpi (*n* = 8). Frequency (%) of small (<400 µm^2^) and large (>1000 µm^2^) myofibers in EDL muscle at (C,D) 8 dpi (*n* = 8) and (G,H) 14 dpi (*n* = 8). Summary values are means ± SEM. Comparisons made by two-way repeated measures ANOVA; *p* < 0.05 = significant; Figure S2: IFNγ at (A) 8 dpi (*n* = 8), (E) 14 dpi (*n* = 8), and (I) serum (*n* = 5–7); IL-4 at (B) 8 dpi (*n* = 6–8), (F) 14 dpi (*n* = 4–7), and (J) serum (*n* = 2–6); IL-5 at (C) 8 dpi (*n* = 8), (G) 14 dpi (*n* = 8), and (K) serum (*n* = 6–7); IL-12p70 at (D) 8 dpi (*n* = 8), (H) 14 dpi (*n* = 8), and (L) serum (*n* = 6–7). Summary values are means ± SEM. Comparisons were made by one-way (serum) or two-way repeated measures ANOVA; *p* < 0.05 = significant.
