# Supplementary material for: An Ion-Releasing Bioactive Glass Accelerates Skeletal Muscle Regeneration and Functional Recovery Following Acute Injury
Source: J Funct Biomater. 2026 Oct 3;17(10):500. doi: 10.3390/jfb17100500 (PMC13706156; doi:10.3390/jfb17100500)
Supplement: Supplementary file 1 [file jfb-17-00500-s001.zip › jfb-4561749-supplementary.pdf]

Supplement Material

Immunoblot Membranes and Total Protein Stains

Pax7

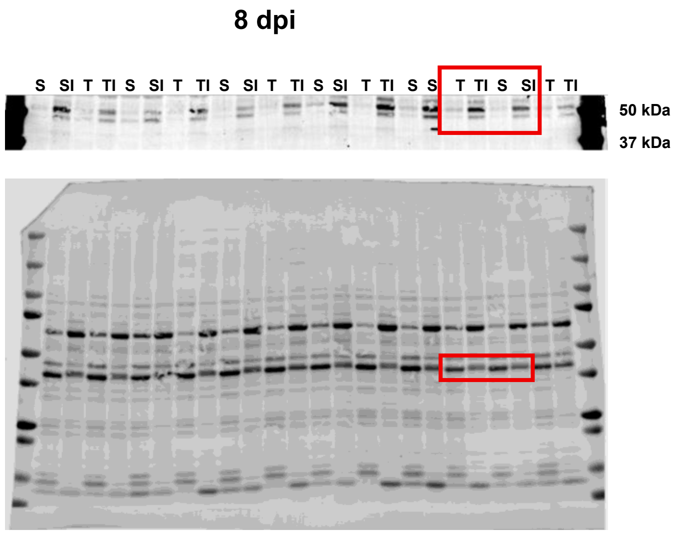

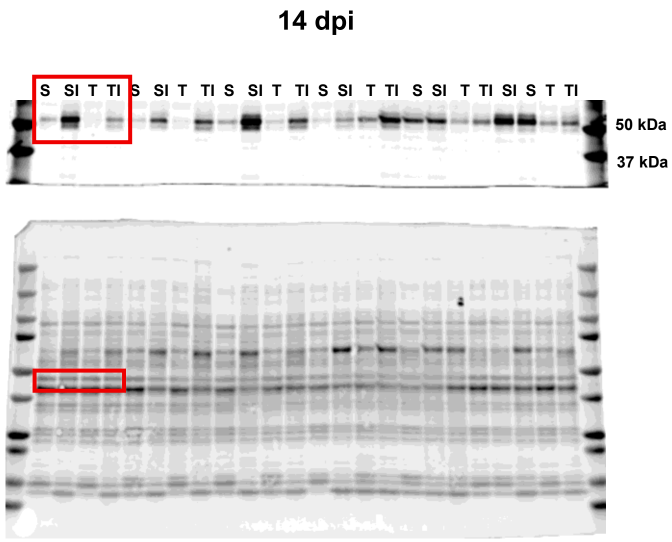

MyoD

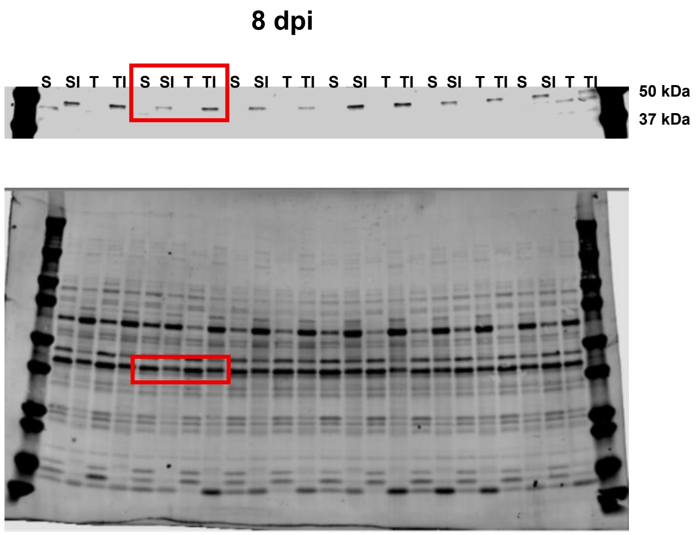

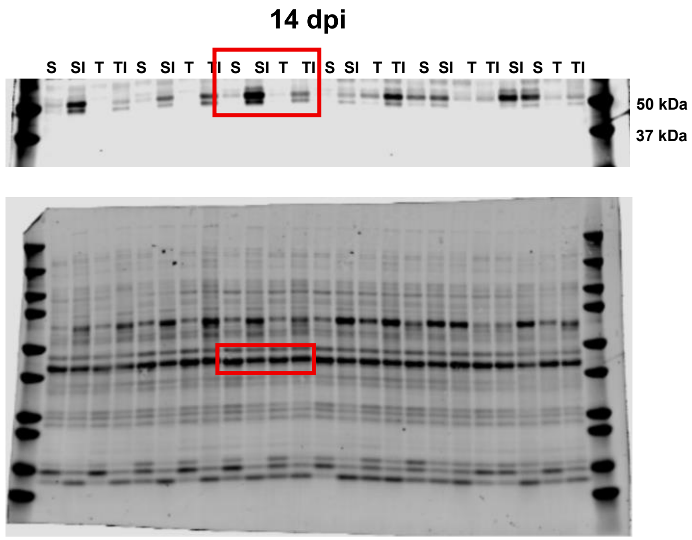

Myogenin

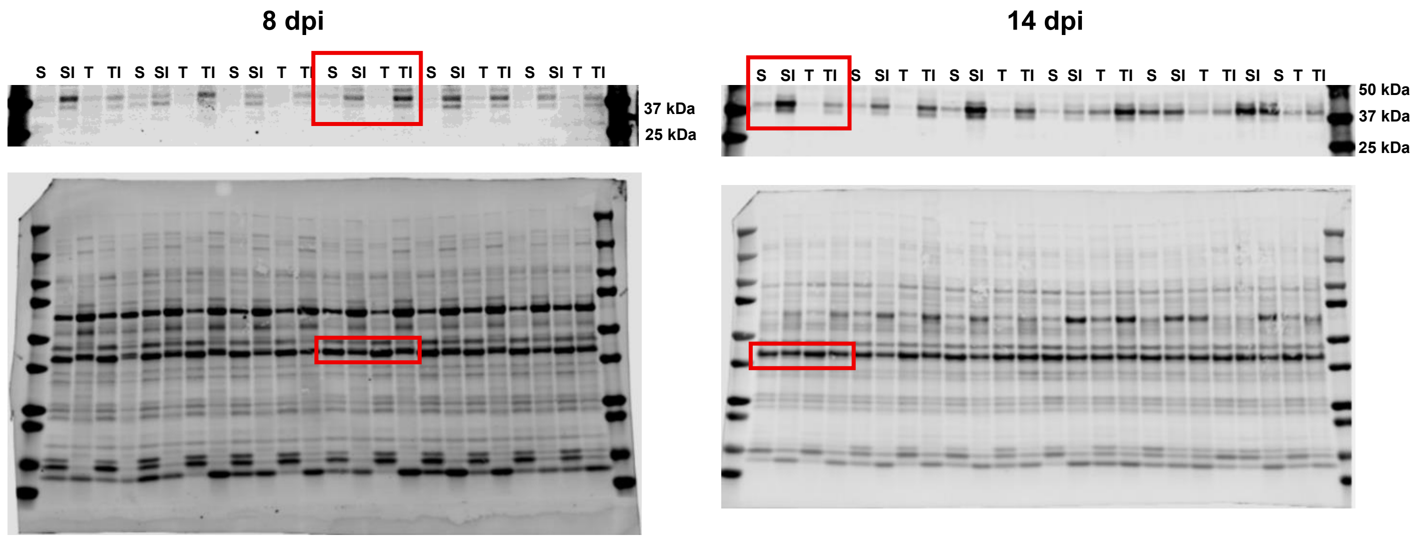
8 dpi
S SI T TI S SI T TI S SI T TI S SI T TI S SI T TI S SI T TI
37 kDa
25 kDa
14 dpi
S SI T TI S SI T TI S SI T TI S SI T TI S SI T TI SI S T TI
50 kDa
37 kDa
25 kDa

Supplement Figure S1

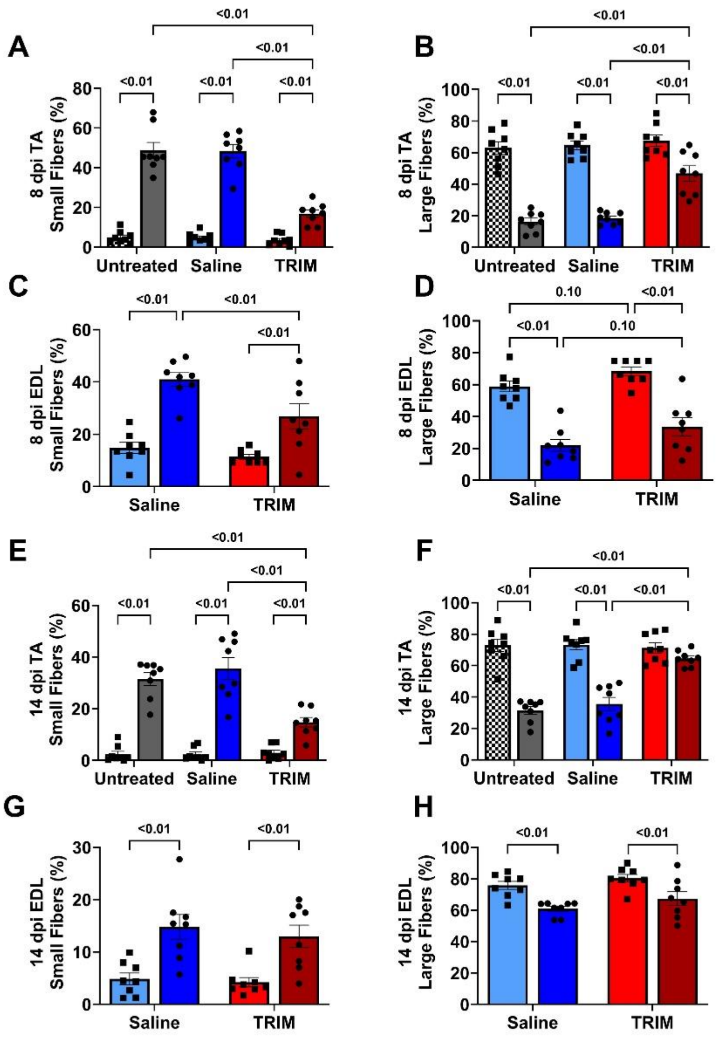

Supplement Figure S1. Frequency (%) of small (<800 μm²) and large (>2000 μm²) myofibers in TA muscle at (A,B) 8 dpi (n = 8) and (E,F) 14 dpi (n = 8). Frequency (%) of small (<400 μm²) and large (>1000 μm²) myofibers in EDL muscle at (C,D) 8 dpi (n = 8) and (G,H) 14 dpi (n = 8). Summary values are means ± SEM. Comparisons made by two-way repeated measures ANOVA; $p < 0.05$ = significant.

Supplement Figure S2

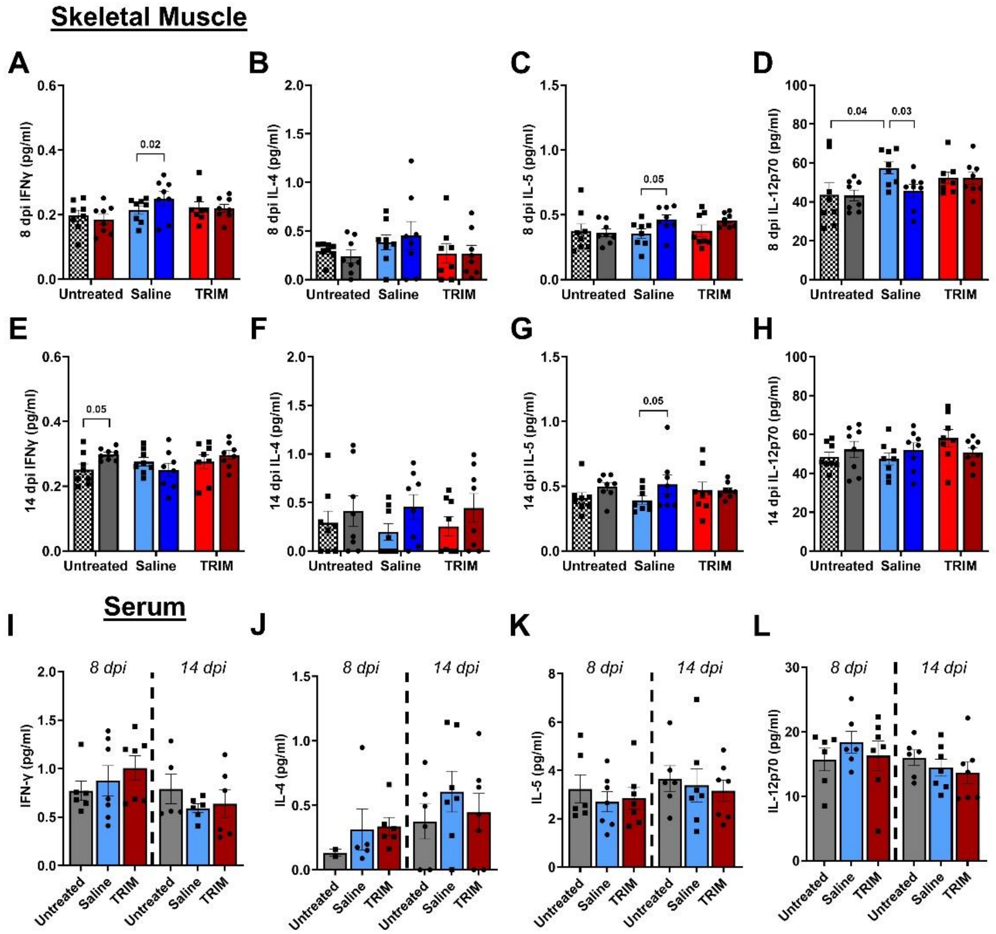

Figure S2. IFNγ at (A) 8 dpi (n = 8), (E) 14 dpi (n = 8), and (I) serum (n = 5–7); IL-4 at (B) 8 dpi (n = 6–8), (F) 14 dpi (n = 4–7), and (J) serum (n = 2–6); IL-5 at (C) 8 dpi (n = 8), (G) 14 dpi (n = 8), and (K) serum (n = 6–7); IL-12p70 at (D) 8 dpi (n = 8), (H) 14 dpi (n = 8), and (L) serum (n = 6–7). Summary values are means ± SEM. Comparisons were made by one-way (serum) or two-way repeated measures ANOVA; $p < 0.05$ = significant.
